# Appraisal of the Neuroprotective Potentials of Isoeugenol Using *In-vitro*, *In-vivo* and *In-silico* Approaches

**DOI:** 10.2174/1570159X22666240329125626

**Published:** 2024-04-24

**Authors:** Bandar A. Alyami, Zeeshan Ahmad, Mehreen Ghufran, Mater H. Mahnashi, Abdul Sadiq, Muhammad Ayaz

**Affiliations:** 1 Department of Pharmaceutical Chemistry, College of Pharmacy, Najran University, Najran, Kingdom of Saudi Arabia;; 2 Department of Pharmacy, Faculty of Biological Sciences, University of Malakand, Chakdara, 18000 Dir (L), KP, Pakistan;; 3 Department of Pathology, MTI Bacha Khan Medical College, Mardan, KP, Pakistan;; 4 Department of Pharmaceutical Chemistry, College of Pharmacy, Najran University, Najran, Kingdom of Saudi Arabia

**Keywords:** Amnesia, Alzheimer’s disease, molecular docking, isoeugenol, Cholinesterase, oxidative stress

## Abstract

**Background:**

Alzheimer's disease (AD) is a neurodegenerative condition that affects the elder population and is linked to behavioral instability and cognitive decline. Only a few drugs are approved for clinical management of AD. Volatile oils and their components exhibit diverse pharmacological potentials, including neuroprotective properties. The current study aimed to evaluate isoeugenol's neuroprotective potentials against cognitive impairments caused by scopolamine.

**Methods:**

Standard protocols were followed in the *in-vitro* antioxidant, cholinesterase inhibitory and molecular docking assays. Isoeugenol was initially evaluated for antioxidant potential using DPPH and ABTS free radicals scavenging assays. Subsequently, AChE/BChE inhibition studies were performed following Ellman’s assay. To assess the compound's binding effectiveness at the enzymes' target site, it was docked against the binding sites of cholinesterase. The effect of isoeugenol supplementation on scopolamine-induced amnesia was assessed using Shallow Water Maze (SWM), Y-Maze and Elevated Plus Maze (EPM) tests.

**Results:**

In DPPH and ABTS assays, isoeugenol exhibited considerable efficacy against free radicals with IC_50_ of 38.97 and 43.76 μg/mL, respectively. Isoeugenol revealed 78.39 ± 0.40% and 67.73 ± 0.03% inhibitions against AChE and BChE, respectively, at 1 mg/ml concentration. In docking studies, isoeugenol exhibited a docking score of -12.2390, forming two hydrogen bonds at the active site residues of AChE. Further, with a docking score of -10.1632, isoeugenol binds adequately to the BChE enzyme *via* two arene-hydrogen interactions and one hydrogen bond.

**Conclusion:**

Isoeugenol offered considerable protection against scopolamine-induced memory deficits and improved the special memory of the rodents.

## 1 INTRODUCTION

Dementia refers to a group of diseases associated with a gradual decline in brain functions, causing cognitive decline, behavioral turbulence and imperfection in routine functions [1]. Alzheimer’s Disease (AD) is the most common cause of dementia among old age population [2]. The disease prominently affects individuals above 65 years and is responsible for 50-60% of all dementia cases. In 2018, about 50 million people were affected by dementia globally, which is estimated to reach 152 million effectors by 2050 [3]. The economic burden of AD is huge, as it is reported that AD costs almost $1 trillion and predicted that it will double by 2030 [4]. AD has a high mortality rate, describing it as just not a disease of memory loss. Neuropathologists revealed various pathological pathways of AD, including the development of β-amyloid plaques (Aβ), neurofibrillary tangles (NFTs), a depletion in cholinergic neurons, a decline in cholinesterase’s and oxidative stress [5, 6]. The Aβ are abnormal deposits of a protein resulting from the breakdown of amyloid precursor protein (APP) by the catalytic action of Beta-Amyloid Precursor Protein (BACE-1), also known as β-secretase [7]. The resulting proteins are sticky in nature and aggregate outside neurons in the brain's parenchyma and cerebral blood vessels and are, therefore, called congophilic angiopathy or cerebral amyloid angiopathy. Deposition of Aβ outside the neurons initiates another pathway inside the neuron, the development of hyperphosphorylated tau proteins which halt transportations inside the neurons [8]. This leads to the loss of cholinergic neurons which brings acetylcholine (ACh) deficiency in the hippocampus, causing memory dysfunctions with impaired cognitive processes. To maintain the cholinergic activities, inhibition of cholinesterase involved in the catalytic breakdown of ACh is among the most viable option now [9]. The synaptic upsurge of ACh relieves behavioral issues and improves memory.

L-(2)-Scopolamine is an alkaloid that competes with acetylcholine for muscarinic receptors, acting as a muscarinic antagonist. Peripherally, it antagonizes parasympathetic nervous system activities, and in the central nervous system, it induces sedation, antiemesis, and amnesia. Scopolamine is structurally similar to atropine, an inhibitor of the parasympathetic system and can be used in conditions requiring decreased parasympathetic activity. It mostly affects the eye, gastrointestinal tract, heart, and exocrine organs [10]. In rodents’ scopolamine inhibits cholinergic neurotransmission and leads to memory and cognition impairments. Current studies have shown that scopolamine also increases ROS accumulation in the brain [11], so it is an ideal agent for inducing amnesia and oxidative stress in rodents.

As the currently available drugs are linked to adverse consequences and poor efficacy, people look back to traditional medicine for treatment, and so far, the plant's products have never disappointed by providing effective remedies with fewer ADRs. Essential oils and their constituents are reported to exhibit diverse pharmacological properties, including efficacy in neurodegenerative disorders [12, 13]. Plant-derived essential oils have numerous lipophilic compounds having a low molecular weight, which can easily cross the blood-brain barrier (BBB) and cell membranes and thus could be one of the best sources for AD therapy [14-16]. A hydroxyphenyl propene known as eugenol, a major constituent of clove oil, has extensive uses in foods and cosmetics. Its claim of traditional use for health benefits was also supported by several scientific reports, such as efficacy against inflammation, oxidative stress and strong antimicrobial potentials. Due to its wide use, the current work was carried out to evaluate the potency of eugenol against AD [17]. Isoeugenol is a plant-derived essential oil that exhibits diverse pharmacological and neuroprotective potentials. For instance, biochemical and behavioral studies suggest that it protects rats against acrylamide-induced neuropathy [18]. It has also been reported that isoeugenol and eugenol ameliorate inflammation, neuronal impairments, and downregulate oxidative stress mediators in the diabetes-induced neuropathy model [19]. However, efficacy in Alzheimer’s disease (AD) using scopolamine-induced amnesia was not reported yet. The current study aimed to evaluate the neuroprotective potentials of isoeugenol against scopolamine-induced cognitive impairments and assessment of its *in-vitro* efficacy against free radicals, cholinesterases and *in-silico* evaluations.

## 2 MATERIALS AND METHODS

### 2.1 Chemicals and Drugs

**Fig. (1):**
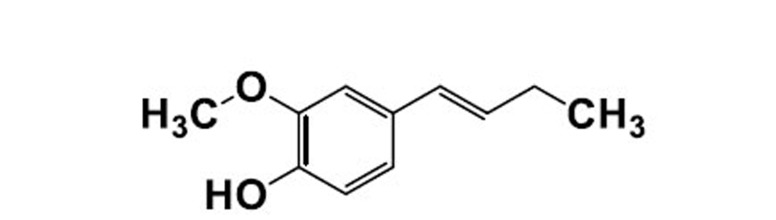
Structure of isoeugenol used in the study.

AChE (type-VI-S, CAS 9000-81-1 Sigma-Aldrich), BChE (CAS 9001-08-5 Sigma-Aldrich), acetylthiocholine iodide (CAS 1866-15-5 Sigma-Aldrich), butyryl thiocholine iodide (CAS 2494-56-6 Sigma-Aldrich), DTNB (CAS 69-78-3 Sigma-Aldrich), Galantamine (CAS 1953-04-4 Siga-Aldrich), DPPH (CAS 1898-66-4 Sigma Aldrich) ABTS (Sigma Aldrich), Methanol (Sigma-Aldrich), Scopolamine (MACKLIN, Lot number C11911980, Cas;6533) were used in the study (Fig. **1**).

### 2.2 Antioxidant Studies

#### 2.2.1 DPPH Free Radical Scavenging Assay

Using the DPPH anti-radicals assay, the antioxidant efficiency of isoeugenol was determined. Following the test sample's serial dilutions (62.5-1000 μg/mL), 1 ml of methanolic DPPH solution was mixed with each sample. The mixture was then left in a dark room for half an hour, and absorbance was taken at 517 nm *via* a spectrophotometer. Vitamin C served as the standard drug in the assay. The following equation was used to estimate the test sample's percent scavenging capacity: (A-B)/A×100, Where the standard drug absorbance is indicated by “A”, and test sample absorbance is indicated by “B” [20].

#### 2.2.2 ABTS Free Radical Scavenging Assay

The anti-radical capacity of isoeugenol was analyzed employing ABTS assay. To the serial dilutions 62.5-1000 μg/mL of isoeugenol, 1 milliliter of methanolic ABTS solution was added, and the mixture was left in a darkened space for half an hour. The absorbance was measured at 745 nm through a spectrophotometer. Again, Vitamin C was used as a standard agent in the assay. Finally, the scavenging potential percentage of the test sample was determined through the formula (A-B)/A×100. A denotes the absorbance of the standard, and B denotes the absorbance of the test sample [21].

### 2.3 Cholinesterase Inhibitory Assay

Eugenol's ability to inhibit cholinesterases was assessed using the AChE and BChE enzymes. The production of 5-thio-2-nitrobenzoate anion was aided by the hydrolysis of acetylcholine iodide and butyrylcholine iodide, respectively, by these two enzymes. When these anions combine with DTNB (5,5'-dithiobis-(2-nitrobenzoic acid), a yellow-colored molecule is produced. The intensity of yellow was used for quantification through a spectrophotometer, which provides data on the anticholinesterase potential of isoeugenol [22].

#### 2.3.1 Preparation of Solutions

Various concentration solutions of isoeugenol (62.5-1000 µg/ml) were made. Enzyme solutions were freshly prepared in a phosphate having a PH value of 8.0, containing 518 units/ml, and similarly, BChE was prepared in a phosphate buffer containing 7-10 units/ml. Then, the units of each enzyme were adjusted to 0.03 U/ml and 0.01 U/ml. Similarly, a substrate for both enzymes was prepared by taking 14.45 mg/ml acetylcholine and 15 mg/ml butyrylcholine in phosphate buffer. All the solutions were stored in the refrigerator. Galantamine was taken and dissolved in methanol and was used as positive control.

#### 2.3.2 Assay procedure & Spectroscopic Analysis

Sample solutions with variable concentrations were mixed with 50 ul enzyme solution, and after the addition of 50 ul DTNB, it was incubated at 37C for 15 min. Each vial was supplemented with 50 ul of substrate after the incubation period, and its absorbance was assessed at 415 nm. For comparison, galantamine, which is a standard AChE inhibitor, was used. The difference in initial absorbance and absorbance after 4 minutes was recorded, and the below equation was used to compute the inhibition percentage [23];

Percentage of enzyme inhibition = Hindered minus percentage of enzyme inhibition;

Percentage of enzyme activity = 100 ×V/Vmax. Here, V represents the rate of change in absorbance over time (ΔAbs/ Δt), and Vmax indicates the peak potency of the enzyme without an inhibitor.

### 2.4 Molecular Docking

The prediction of the binding of compounds to specific proteins is done by a sort of computational modeling called docking studies. They are frequently employed in the research and development of pharmaceuticals to find prospective substances that could bind to a given target protein and alter its function. The molecular docking was carried out using the MOE-Dock tool (www.chemcomp.com) to ascertain the isoeugenol binding interactions at the active site of the enzyme AChE and BChE [24]. These enzymes' 3D crystal structures have been retrieved from the Protein Databank (PDB) along with their PDB IDs, 4M0E (AChE) and 1P0P (BChE). The Molecular Operating Environment (MOE) software was utilized to eliminate every water molecule from the recovered crystal structures. Following 3D protonation to introduce hydrogen atoms to the protein structures, minimization of energy was performed *via* MOE standard configurations. The isoeugenol structure was constructed using these default parameters, and energy was reduced. Then, isoeugenol was docked into the target enzymes' active sites in MOE (www.chemcomp.com). *i.e*., Triangle Matcher placement, London dG scoring first, Induced Fit refinement, Forcefield default, London dG scoring second. Ten conformations were produced for each ligand, and the conformation with the maximum docking score was subjected to additional molecular docking. Using MOE software, the optimal postures with polar, H-pi, and pi-H interactions were examined following molecular docking [25].

### 2.5 *In-vivo* Assays

#### 2.5.1 Animal grouping and Amnesia Induction

This research was evaluated and approved by the Departmental Ethics Committee (DREC) at the Department of Pharmacy University of Malakand with ref No. DREC/Pharm/12-2023. BALB/c mice, mixed breed (Both genders), weighing 25-30 g, were provided with natural light/dark conditions and access to food and water *ad libitum*. For induction of amnesia in mice, scopolamine was used and given (1 mg/kg) intraperitoneally for 9 consecutive days. The mice were divided into four groups:

- Normal/Saline treated group.
- Disease/Scopolamine (1 mg/kg) treated group.
- Standard/Galantamine (8 mg/kg) + Scopolamine (1 mg/kg i/p) treated group.
- Test/Isoeugenol (1 mg/kg) + Scopolamine (1 mg/kg) treated group.

With the exception of the normal group, scopolamine was given to each of these groups [26]. Animals went through an acclimatization period of 5 days, followed by induction of amnesia by I/P administration of scopolamine for 9 days, followed by training of all groups for 9 days. Finally, standard (8 mg/kg) and test isoeugenol (1 mg/kg) were administered (I/P) for 7 days, and tests were conducted [25], as summarized in Fig. (**2**).

### 2.6 Shallow Water Maze Test

The paddling pool model having an octangular design was used. The test was carried out through the Robert M.J. Deacon protocol. The model consisted of a grey base containing shallow water which is bounded by transparent acrylic plastic walls. The model has an 86 cm diameter and contains 8 exits; among them, one exit is open and connected to a pipe and the remaining exits are closed with black plastic plugs. The temperature of water was 20°C to 25°C to provide comfort to the mice and also eliminate temperature base stimuli that could disturb the final result. The experimental room also contained some external cues, such as pictures and lamps which act as orientation for the experimental animals. These cues were kept consistent throughout the experimental procedure [27]. Before the injection of the drug, all groups of mice undergo 9-day training trials. Animals were positioned in the middle of the pool at various directions to the escape tube and the training session lasted for 60 seconds. Most of the mice reached the escape tube quickly; the mice that failed to reach the escape tube were guided manually, and the escape time was measured as escape latency in seconds. Animals of the disease group were confined untreated, while the normal group animals received saline. The standard group consisted of mice receiving galantamine, and the test group to whom the test sample was given at a dosage of 1 mg/kg/day for 7 days successively. Finally, animals were subjected to testing, and their escape latency was measured [27].

### 2.7 Spontaneous Alternation Y-Maze Test

Utilizing the Y-maze model, the short-term memory of the rodents was assessed. The primary goal of this model was to assess spatial working memory, which involves the interaction of several brain regions, including the frontal cortex and hippocampus. The three arms of the Y-maze are positioned at 120-degree angles to one another. The animals were allowed to roam around and investigate the maze on their own. Even when they have visited the previously visited part, mice with high recall will enter the less explored arm. Mice's ability to recall and retain spatial knowledge is demonstrated by their behavior. The procedure uses the mice's natural curiosity to explore new places [28]. The mice were trained for nine days, and at the start of the trials, they were acclimated to the trial compartment. Initially, 70% propanol was used to disinfect the area before the trial began. The maze's labyrinth had three arms, designated A, B, and C. The animals were positioned in arm A, facing the center of the maze, and were free to walk around and explore the maze while an eight-minute recording was conducted. After 8 minutes, the mouse was carefully returned to its cage, and again, the maze was sanitized with 70% propanol prior to the introduction of the new mouse. After completion of the trial, the number of each arm’s entries was counted and alternation was assessed, which designated the spatial memory of the mouse [29]. The formula to calculate the percentage alternation was as follows:

Percentage of alternation = (No of Alternations / Total No of arm entries - 2) ×100.

### 2.8 Elevated Plus Maze (EPM) Test

Retention and gaining of memory of the animals (mice) were assessed through the EPM model. The model is in plus shape with four arms; in them, two were open (35×5 cm), and two were closed (35×5×15 cm) and had 25 cm height. The testing room was provided with dim light, and the trials were carried out between 10 am and 4 pm for nine consecutive days. As we previously mentioned, animals went through training sessions for 9 consecutive days. The mice were placed in the experimental room one hour before the test in order to acclimatize to the experimental room. The video recorder was kept above the maze and a mouse was carefully placed on the open arm of the maze, facing opposite to the center of the maze. The time in which the mouse enters into the closed arm is termed as transfer latency and was recorded during the trials, and the footage was evaluated to calculate the transfer latency and memory acquisition of mice.

### 2.9 Statistical Analysis

Data was calculated as mean ± SEM. Figures were generated *via* GraphPad Prism and IC_50_ were determined from dose-response data. One-way ANOVA followed by multiple comparisons Dunnett’s test was applied to the results for comparisons of test groups with standards. A *p*-value of less than 0.05 was deemed statistically significant.

## 3 RESULTS AND DISCUSSION

### 3.1 Isoeugenol Exhibits Scavenging effects against DPPH Radicals

In the DPPH assay, Isoeugenol demonstrated a concentration-dependent pattern of percent inhibition against ABTS free radicals, as shown in Fig. (**3**). It demonstrated 83.51 ± 0.54, 75.76 ± 0.61, 67.22 ± 1.28, 63.51 ± 0.54 and 56.37 ± 0.56 percent inhibition against DPPH free radicals at 1000, 500, 250, 125 and 62.5 μg/mL concentrations correspondingly and an IC_50_ of 38.97 μg/mL. Further, % DPPH inhibition of isoeugenol was comparable with gallic acid, having IC_50_ of 13.72 μg/mL.

### 3.2 Inhibitory Study against ABTS Radicals

**Fig. (4):**
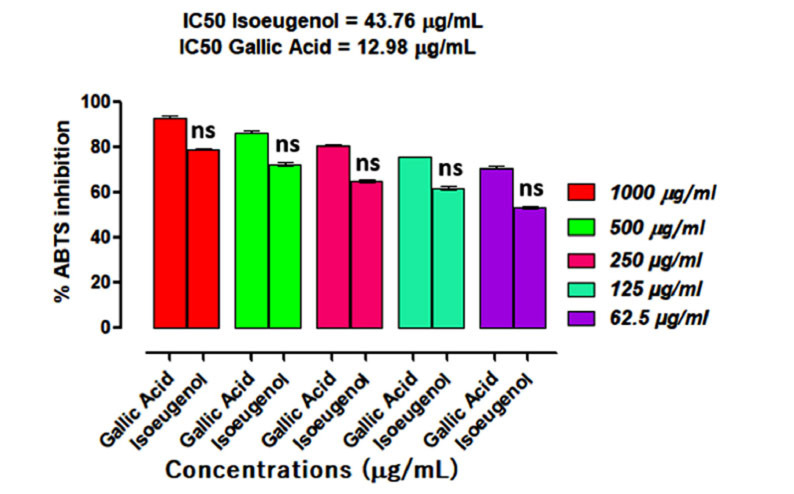
Results of isoeugenol inhibitory potentials against ABTS radicals. Results were expressed as a Mean ± SEM of three experimental observations. Ns represent results that were not significantly different (*p* > 0.05) when compared with positive control (Gallic acid) at the same tested concentrations.

Isoeugenol demonstrated a high % inhibition against ABTS free radicals in a dose-dependent way (Fig. **4**). It demonstrated 79.08 ± 0.47, 72.56 ± 1.06, 65.03 ± 0.35, 61.90 ± 1.55 and 53.42 ± 0.46 percent inhibition at the same concentrations mentioned above and IC_50_ of 43.76 μg/mL. Isoeugenol percent scavenging was comparable with gallic acid, whose IC_50_ value was 12.98 μg/mL.

### 3.3 AChE Inhibition Study

In the AChE inhibition assay, the compound isoeugenol showed concentration-dependent AChE inhibition, as shown in Fig. (**5**). It demonstrated 78.39 ± 0.40, 54.29 ± 0.32, 45.34 ± 0.35, 34.02 ± 0.24 and 26.35 ± 0.11 percent inhibition at 1000, 500, 250, 125 and 62.5 μg/mL concentrations, respectively, with IC_50_ of 430.42 μg/mL in comparison to galantamine (positive control), whose IC_50_ value was 30.54 μg/mL.

### 3.4 BChE Inhibition Study

In the BChE inhibition assay, the compound isoeugenol demonstrated BChE inhibition in a dose-dependent way, as shown in Fig. (**6**). Our sample demonstrated 67.73 ± 0.03, 57.42 ± 0.12, 47.39 ± 0.35, 41.36 ± 0.71 and 29.15 ± 0.22 percent inhibition at 1000, 500, 250, 125 and 62.5 μg/mL concentrations and IC_50_ of 277.91 μg/mL in comparison to galantamine (positive control), whose IC_50_ value was 21.30 μg/mL.

### 3.5 Molecular Docking Analysis

**Fig. (7):**
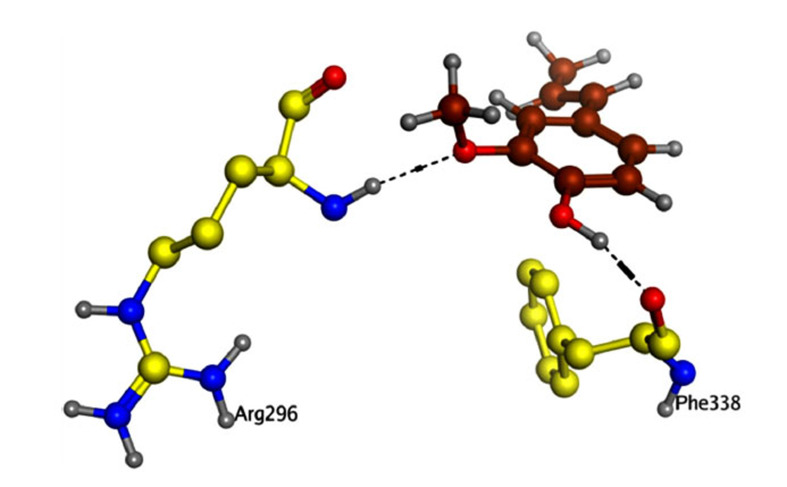
Isoeugenol's binding mode to the active residues of AChE.

**Fig. (8):**
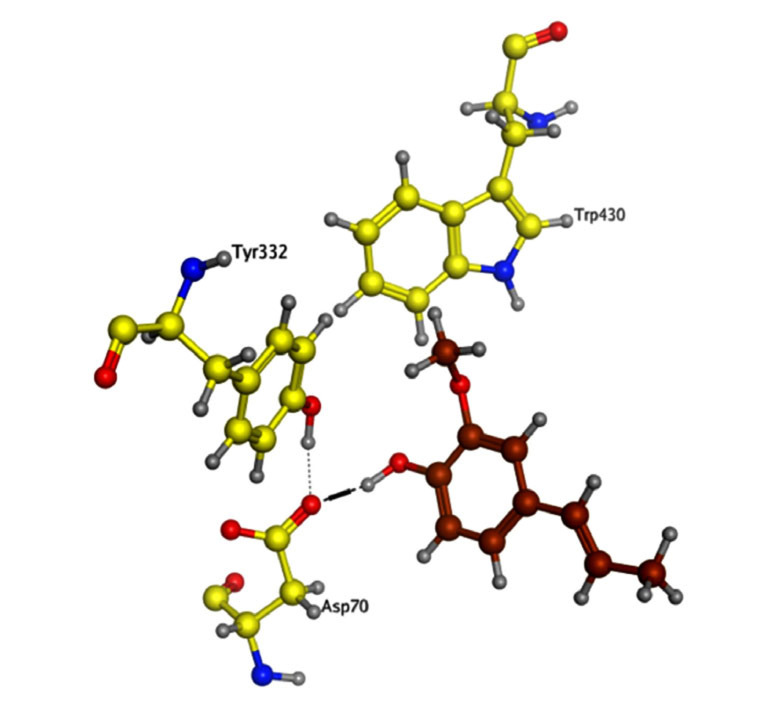
Binding mode of compound isoeugenol with the active residues of BChE.

The interaction of isoeugenol with the AChE/BChE was examined utilizing a molecular docking approach. The isoeugenol was properly accommodated in the target enzymes' active sites. The isoeugenol (docking score = -12.2390) was shown to have created two hydrogen bonds with the AChE active site residues based on its docking configuration (Fig. **7**). Isoeugenol exhibited a docking score of -10.1632 against BChE and is effectively bonded to the BChE enzyme through two arene-hydrogen interactions and one hydrogen bond (Fig. **8**).

### 3.6 Behavioral Studies

#### 3.6.1 Isoeugenol offers Protection against Cognitive Impairments in the SWM Paradigm

**Fig. (9):**
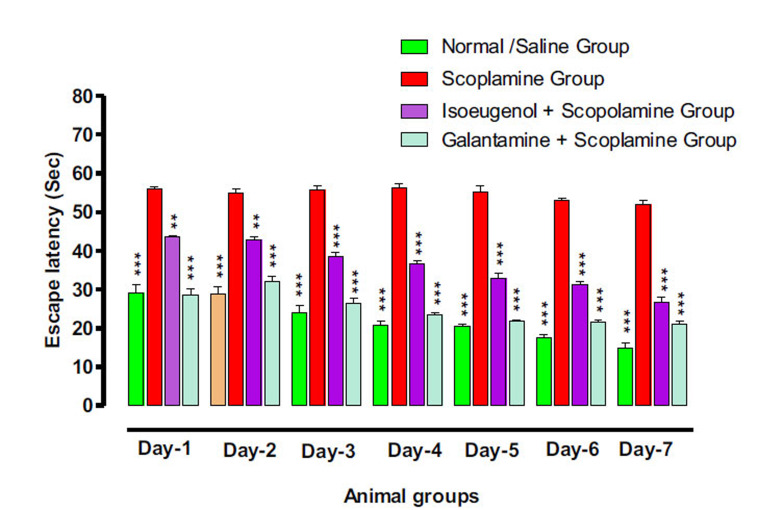
Effect of isoeugenol on transfer latency of animals in SWM test. Results indicate the Mean ± SEM of three observations. One-way ANOVA followed by multiple comparisons Dunnett’s test was applied to the results for comparisons of test groups with standards. ** = *p* < 0.01 and *** *p* < 0.001 when compared with Galantamine treated groups.

Using a shallow water maze test, the mice's spatial working memory was assessed. Even after nine days of training, untreated amnesic animals show a high latency time indicating poor working memory of mice with an average escape latency of 54.23 sec from day 1 to 7 (Fig. **9**). At the same time, the mice in the standard group showed substantial improvement in their working memory with an average escape latency of 23.34 sec. The mice in the test group have a slight improvement in their working memory on the first four days with an escape latency of 43.86 sec, but after day 5, the mice showed significant improvement in the memory with average escape latency of 36.57 sec, 32.99 and eventually 26.62 sec on day 6 and 7. As expected the mice in the control group have minimal latency of 19.18 sec from day 1 to day 7.

#### 3.6.2 Isoeugenol Improved Spontaneous Alternation Behavior in the Y-Maze Test

**Fig. (10):**
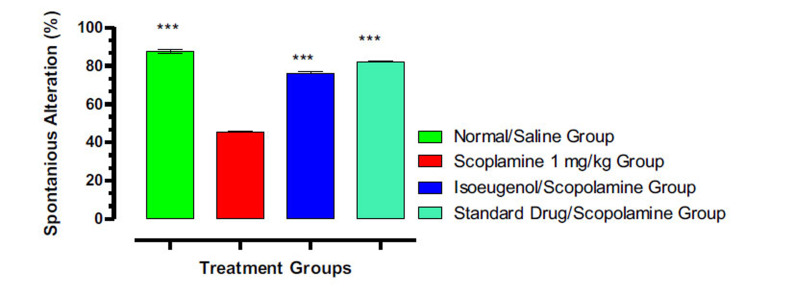
Effect of isoeugenol on animal's special working memory in the Y-Maze test. Results indicate the Mean ± SEM of three observations. One-way ANOVA followed by multiple comparison Dunnett’s test was applied to the results for comparisons of test groups with standards. *** *p* < 0.001 when compared with the scopolamine-treated group.

Results of the Y-maze procedure were expressed as percent spontaneous alternations, which designate the special working memory of mice (Fig. **10**). Those mice that have shown a high frequency of alternation have the highest cognition abilities and display less damage to the memories. Disease animals have the least special working memory with 45% alternation, while the mice in the control and isoeugenol-treated groups have shown the highest working memory with 82.56% and 76.10% alternation, respectively, when compared with disease animals. However, the mice in the normal group have the highest percent of 87.74 among all groups.

#### 3.6.3 Results of the EPM Test

The elevated plus maze model was used to elaborate the animal’s learning and memory retention capacity. The results of EPM were written in Transfer latency time (TL). The TL on the first day of the training session specifies the learning ability of mice, while the TL on the next following days shows the retention of memory, as illustrated in Fig. (**11**). The TL of the normal control group on the 7^th^ and 8^th^ day was 40.36 and 28.93 sec, respectively. While the mice in the disease group exhibit an increase in TL 49.57 and 60.23 on days 7^th^ and 8^th^, which shows impaired working memory. Animals of galantamine and isoeugenol-treated groups have shown decreased TL on days 7^th^ and 8^th^, which indicates the improvement in the memory and learning ability of mice. The TL of eugenol-treated mice was 51.64 and 41.12 sec, respectively. While the TL standard administers, the group was 47.82 and 36.89, respectively.

## 4 DISCUSSION

Aging is linked with numerous physiological changes in the brain, which include a decline in cognitive functions, synaptic dysfunctions and loss of memory [30]. Particularly, individuals having age above 65 are at high risk of developing brain dysfunctions such as AD [31]. Appropriate nutritional interventions, drug therapies and alternative therapies are currently under consideration. Medicinal plants are a rich source of bioactive molecules and have been in use since antiquity [32]. Plants-derived essential oils are low molecular mass compounds that can cross the cell membrane and blood-brain barrier effectively and could be the best options for the management of brain anomalies like AD and Parkinson's disease [33]. As we know, ACh, once released at the synaptic cleft, causes impulse transmission across the synapse, and its action is terminated by the catalytic action of the enzyme cholinesterase [22]. However, in AD patients’ brain, there is a loss of cholinergic neurons, which causes inadequate production of ACh, causing a depletion in ACh level and thus memory impairments arise. Therefore, AChE/BChE inhibitors are administered, which increases the ACh synaptic level *via* inhibition of its metabolizing enzymes. Galantamine, rivastigmine, and donepezil are AChE inhibitors permitted by the FDA for AD treatment [12]. However, these drugs are linked with some severe ADRs like hepatotoxicity and nephrotoxicity and searching for novel drugs having the least ADRs and high efficacy is still a challenge in the medical world. Many plant products have been studied and their studies revealed the presence of certain phytochemicals that inhibit cholinesterase effectively. Additional research on these plant products may result in the development of a novel medication for the therapy of AD [34]. In this current study, we found that eugenol has effectively inhibited AChE/BChE enzymes. The AChE percent inhibition of eugenol at different concentrations (62.5-1000 µg/ml) was 78.39 ± 0.40, 54.29 ± 0.32, 45.34 ± 0.35, 34.02 ± 0.24 and 26.35 ± 0.11 correspondingly, and IC_50_ of 430.42 μg/mL. Whereas, galantamine (positive control) revealed an IC_50_ value of 30.54 μg/mL. Regarding BChE inhibition activity eugenol exhibit 67.73 ± 0.03, 57.42 ± 0.12, 47.39 ± 0.35, 41.36 ± 0.71 and 29.15 ± 0.22 percent inhibition at 1000, 500, 250, 125 and 62.5 μg/mL concentrations respectively with IC_50_ of 277.91 μg/mL in comparison to galantamine (positive control), whose IC_50_ value was 21.30 μg/mL.

Free radicals are linked with numerous human diseases, such as cancer, arthritis, CNS damage, and neurodegenerative disorders [35]. The body produces these free radicals during the metabolic processes, but these radicals are scavenged by antioxidants in the body such as glutathione-reductase, catalase and hydroxylase. When there is a depletion of immune system antioxidants in the body or in immunocompromised individuals, these radicals cause harm to proteins, nucleic acids, and enzymes, leading to the generation of oncogenes. Neurons are more vulnerable to oxidation because of more oxygen consumption. Due to oxidations in the brain, there is an increased level of abnormal proteins, lipids, carbohydrates, and DNA, especially in those areas that have high Aβ concentrations [36]. Oxidative stress also upregulates proinflammatory cytokines and causes permanent DNA damage [37]. Both synthetic and natural antioxidants are available, which protect the immune system from the injuries of oxidation [38, 39]. A few of the synthesized antioxidants, like gallic acid, tertiary butylated hydroquinone, butylated hydroxyanisole (BHA), and butylated hydroxytoluene (BHT), are used, but they have many side effects. Conversely, natural antioxidants have fewer side effects and are considered safer alternatives, such as resveratrol, quercetin and epigallocatechin-3-gallate (EGCG) [40-42]. In our current study, isoeugenol was effective against both DPPH/ABTS radicals with an IC_50_ of 38.97 μg/mL. Our compound was considerably effective against ABTS radicals with IC_50_ of 43.76 μg/mL. So, this natural antioxidant having cholinesterase inhibitory potentials might be useful in the management of neurodegenerative diseases.

*In-silico* modeling of the potential compounds against the active binding site of the target protein is among the key aspects during the preliminary stages of drug discovery [43, 44]. Molecular docking provides useful information about the binding energy of the compounds against the target sites and hence provides information about their binding affinity with the biological targets [45, 46]. It helps researchers to sort potential bioactive compounds among huge lots of molecules, thus saving precious time and energy and reducing research expenditures. *In-vitro* enzyme inhibition assays, coupled with molecular docking studies, help researchers select compounds for details animal studies on rational grounds. Keeping the same in mind, after *in-vitro* evaluation of isoeugenol against free radicals and AChE/BChE enzymes, molecular docking studies were performed. We observed that isoeugenol was effectively accommodated at the target site of the enzymes with docking score = -12.2390 for AChE forming two hydrogen bonds. Further, it was bound with BChE *via* two arene-hydrogen interactions and one hydrogen bond and exhibited a docking score of -10.1632. The binding energy indicates considerable affinity of the isoeugenol with both enzymes.

In neuroscience, scopolamine is mostly utilized to induce memories and cognitive impairments in experimental models (such as rodents) because it can easily cross the blood-brain barrier. In AD studies, scopolamine can cause cholinergic dysfunction and also increase amyloid-β deposition between the neurons; both are the hallmark pathological pathways of AD. Therefore, the use of scopolamine in AD research is significant in identifying new ways of treating the disease [47]. Several behavioral models were used to evaluate the mice's response to treatment upon induction of amnesia in mice. The SWM apparatus is used to evaluate the learning ability and cognitive functions of mice. The apparatus comprised of white base and transparent walls. The walls contain eight exits, of which one exit was kept open while the rest were closed with plastic plugs. The base of the pool is provided with shallow water of about 2 cm, which serves as a stimulus for mice. At the end of the training session and treatment with isoeugenol, we concluded that the escape latency was close to the latency of galantamine and gradual improvement in memories was observed from day 1 to day 7. In the case of the Y maze test, isoeugenol has significantly improved the short-term memory and cognitive functions of mice, which is comparable to the behavior (spontaneous alternation) of mice treated with galantamine and saline [48]. The ability of mice to retain and recall memory was evaluated in the EPM test. We observed that the transfer latency time of mice treated with isoeugenol was lower than animals of the disease group and comparable to standard group and saline group results [49]. Further studies such as immunohistochemistry and antioxidant biomarkers analysis in the brain are required.

## CONCLUSION

In the current study, isoeugenol exhibited considerable cholinesterase inhibitory and antioxidant properties. The compound fit well in the target site of the enzymes and exhibited considerable binding affinity for the target proteins. The antioxidant potentials coupled with cholinesterase inhibitory results might be very useful against dual targets of AD. Behavioral studies revealed the neurocognitive benefits of the tested samples in animal models of amnesia. Though the isoeugenol dose was very low in the behavioral studies as compared to the standard drug, considerable improvement was observed. Chronic neuroprotective studies at high doses supplemented by molecular studies are warranted to assess the effect of isoeugenol on amyloid proteins and tauopathies.

## AUTHORS’ CONTRIBUTIONS

It is hereby acknowledged that all authors have accepted responsibility for the manuscript's content and consented to its submission. They have meticulously reviewed all results and unanimously approved the final version of the manuscript.

## Figures and Tables

**Fig. (2):**
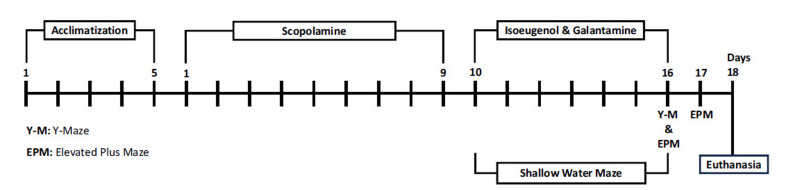
Study scheme for the *in-vivo* experiments.

**Fig. (3):**
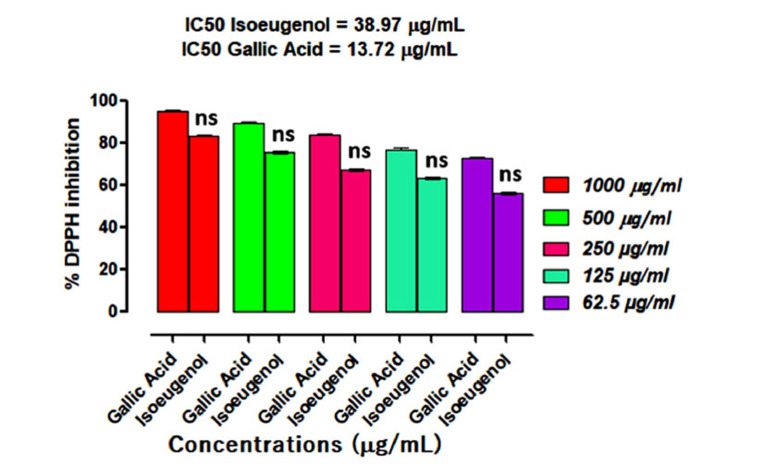
Results of isoeugenol inhibitory potentials against DPPH radicals. Results were expressed as a Mean ± SEM of three experimental observations. Ns represent results that were not significantly different (*p* > 0.05) when compared with positive control at the same tested concentrations.

**Fig. (5):**
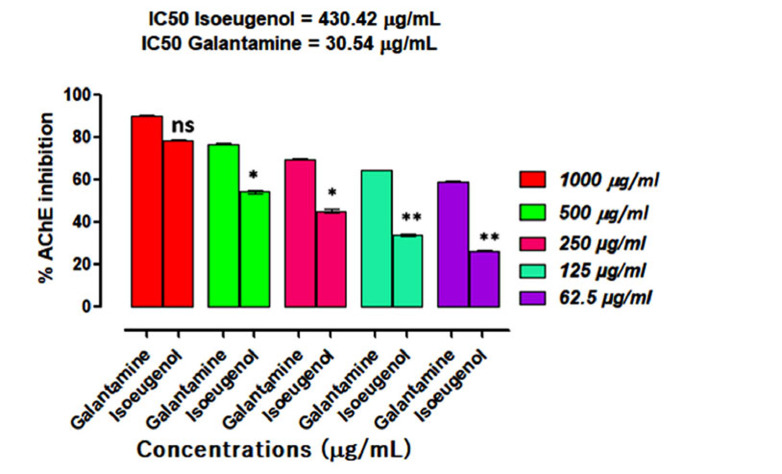
Results of isoeugenol inhibitory potentials against AChE. Results indicate the Mean ± SEM of three observations. One-way ANOVA followed by multiple comparisons Dunnett’s test was applied to the results for comparisons of test groups with standards. Ns = *p* > 0.05, * = *p* < 0.05 and ** *p* < 0.01 when compared with positive control (Galantamine) at the same tested concentrations.

**Fig. (6):**
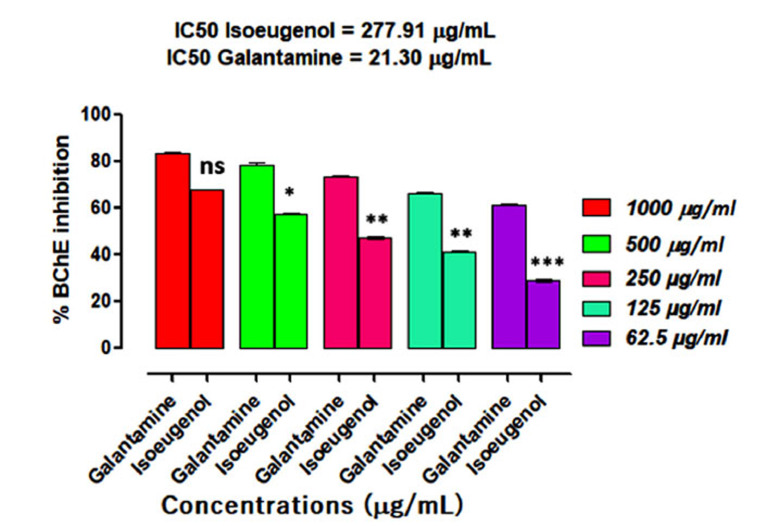
Results of isoeugenol inhibitory potentials against BChE. Results indicate the Mean ± SEM of three observations. One-way ANOVA followed by multiple comparisons Dunnett’s test was applied to the results for comparisons of test groups with standards. Ns = *p* > 0.05, * = *p* < 0.05, ** = *p* < 0.01 and *** *p* < 0.001 when compared with positive control (Galantamine) at the same tested concentrations.

**Fig. (11):**
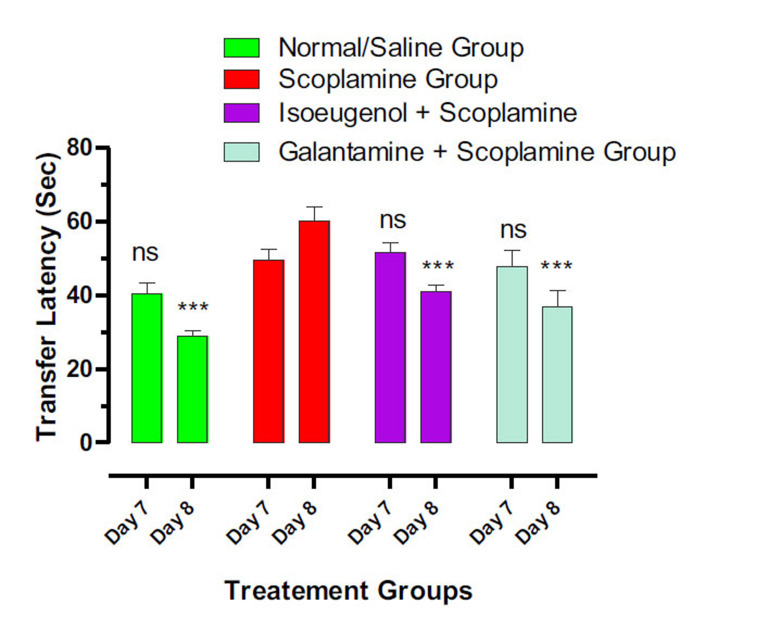
Effects of eugenol on the animal’s transfer latency in EPM test. Results indicate the Mean ± SEM of three observations. One-way ANOVA followed by multiple comparison Dunnett’s test was applied to the results for comparisons of test groups with standards. ns = *p* > 0.05 and *** *p* < 0.001 when compared with scopolamine-treated amnesic animals’ groups.

## Data Availability

The data that support the findings of this study are available from the corresponding author, [M.A.], upon reasonable request.

## LIST OF ABBREVIATIONS

ACh: Acetylcholine
AD: Alzheimer’s Disease
APP: Amyloid Precursor Protein
BBB: Blood-brain Barrier
BHA: Butylated Hydroxyanisole
BHT: Butylated Hydroxytoluene
EGCG: Epigallocatechin-3-gallate
MOE: Molecular Operating Environment
NFTs: Neurofibrillary Tangles
PDB: Protein Databank

## References

[1] Alam W., Hussain Y., Ahmad S., Ali A., Khan H. (2023). In: Phytonutrients and Neurological Disorders Therapeutic and Toxicological Aspects..

[2] Alharthy K., Balaha M., Devi S., Altharawi A., Yusufoglu H., Aldossari R., Alam A., Giacomo D.V. (2023). Ameliorative effects of isoeugenol and eugenol against impaired nerve function and inflammatory and oxidative mediators in diabetic neuropathic rats.. Biomedicines.

[3] (2016). Alzheimer’s Association. 2016 Alzheimer’s disease facts and figures.. Alzheimers Dement..

[4] Ayaz M., Junaid M., Ullah F., Subhan F., Sadiq A., Ali G., Ovais M., Shahid M., Ahmad A., Wadood A., El-Shazly M., Ahmad N., Ahmad S. (2017). Anti-Alzheimer’s studies on β-sitosterol isolated from Polygonum hydropiper L.. Front. Pharmacol..

[5] Ayaz M., Nawaz A., Naz F., Ullah F., Sadiq A., Ul Islam Z. (2022). Phytochemicals-based therapeutics against Alzheimer’s disease: An update.. Curr. Top. Med. Chem..

[6] Bai X., Yan C., Yang G., Lu P., Ma D., Sun L., Zhou R., Scheres S.H.W., Shi Y. (2015). An atomic structure of human γ-secretase.. Nature.

[7] Butterfield D.A., Griffin S., Munch G., Pasinetti G.M. (2002). Amyloid β-peptide and amyloid pathology are central to the oxidative stress and inflammatory cascades under which Alzheimer’s disease brain exists.. J. Alzheimers Dis..

[8] Chang C.C., Chang C-Y., Huang J-P., Hung L-M. (2012). Effect of resveratrol on oxidative and inflammatory stress in liver and spleen of streptozotocin-induced type 1 diabetic rats.. Chin. J. Physiol..

[9] Chen W.N., Yeong K.Y. (2020). Scopolamine, a toxin-induced experimental model, used for research in Alzheimer’s disease.. CNS Neurol. Disord. Drug Targets.

[10] Colombres M., Sagal J., Inestrosa N. (2004). An overview of the current and novel drugs for Alzheimer’s disease with particular reference to anti-cholinesterase compounds.. Curr. Pharm. Des..

[11] da Costa I.M., Pedrosa E.C.G.A., de Bezerra C.A.P., Fernandes L.C.B., de Cavalcanti P.J.R.L., Freire M.A.M., de Araújo D.P., do Rego A.C.M., Filho A.I., Pinheiro F.I. (2020). In: Neuroprotection- New Approaches and Prospects; IntechOpen,.

[12] Deacon R.M. (2013). Shallow water (paddling) variants of water maze tests in mice.. J. Vis. Exp..

[13] Ellman G.L., Courtney K.D., Andres V., Featherstone R.M. (1961). A new and rapid colorimetric determination of acetylcholinesterase activity.. Biochem. Pharmacol..

[14] Ghufran M., Ullah M., Khan H.A., Ghufran S., Ayaz M., Siddiq M., Abbas S.Q., Hassan S.S., Bungau S. (2023). In-silico lead druggable compounds identification against SARS COVID-19 main protease target from in-house, chembridge and zinc databases by structure-based virtual screening, molecular docking and molecular dynamics simulations.. Bioengineering.

[15] Golechha M., Bhatia J., Arya D.S. (2012). Studies on effects of Emblica officinalis (Amla) on oxidative stress and cholinergic function in scopolamine induced amnesia in mice.. J. Environ. Biol..

[16] Halliwell B. (1994). Free radicals, antioxidants, and human disease: Curiosity, cause, or consequence?. Lancet.

[17] Bagci E., Aydin E., Mihasan M., Maniu C., Hritcu L. (2016). Anxiolytic and antidepressant-like effects of Ferulago angulata essential oil in the scopolamine rat model of Alzheimer’s disease.. Flavour Fragrance J..

[18] Kern S., Zetterberg H., Kern J., Zettergren A., Waern M., Höglund K., Andreasson U., Wetterberg H., Börjesson-Hanson A., Blennow K., Skoog I. (2018). Prevalence of preclinical Alzheimer disease.. Neurology.

[19] Kraeuter A-K., Guest P.C., Sarnyai Z. (2019). In: Preclinical
models: Techniques and protocols; Humana Press: New
York, NY,.

[20] Kühnau J. (1976). The flavonoids. A class of semi-essential food components: their role in human nutrition.. World Rev. Nutr. Diet..

[21] Kumar A., Singh A. (2015). Ekavali, A review on Alzheimer’s disease pathophysiology and its management: An update.. Pharmacol. Rep..

[22] Kumpulainen J.T., Salonen J.T. (1999). Natural Antioxidants and Anticarcinogens in Nutrition, Health and Disease..

[23] Lee J.S., Kim H.G., Lee H.W., Han J.M., Lee S.K., Kim D.W., Saravanakumar A., Son C.G. (2015). Hippocampal memory enhancing activity of pine needle extract against scopolamine-induced amnesia in a mouse model.. Sci. Rep..

[24] Lombardo S., Maskos U. (2015). Role of the nicotinic acetylcholine receptor in Alzheimer’s disease pathology and treatment.. Neuropharmacology,.

[25] Maggio A., Rosselli S., Bruno M. (2016). Essential oils and pure volatile compounds as potential drugs in Alzheimer’s disease therapy: An updated review of the literature.. Curr. Pharm. Des..

[26] Mahnashi M.H., Ashraf M., Alhasaniah A.H., Ullah H., Zeb A., Ghufran M., Fahad S., Ayaz M., Daglia M. (2023). Polyphenol-enriched Desmodium elegans DC. ameliorate scopolamine-induced amnesia in animal model of Alzheimer’s disease: *In vitro*, *in vivo* and *in silico* approaches.. Biomed. Pharmacother..

[27] Mahnashi M.H., Ayaz M., Alqahtani Y.S., Alyami B.A., Shahid M., Alqahtani O., Kabrah S.M., Zeb A., Ullah F., Sadiq A. (2023). Quantitative-HPLC-DAD polyphenols analysis, anxiolytic and cognition enhancing potentials of Sorbaria tomentosa Lindl.. Rehder. J. Ethnopharmacol..

[28] Mahnashi M.H., Ayaz M., Ghufran M., Almazni I.A., Alqahtani O., Alyami B.A., Alqahtani Y.S., Khan H.A., Sadiq A., Waqas M. (2023). Phytochemicals-based β-amyloid cleaving enzyme-1 and MAOB inhibitors for the treatment of Alzheimer’s disease: Molecular simulations-based predictions.. J. Biomol. Struct. Dyn..

[29] Marchese A., Barbieri R., Coppo E., Orhan I.E., Daglia M., Nabavi S.F., Izadi M., Abdollahi M., Nabavi S.M., Ajami M. (2017). Antimicrobial activity of eugenol and essential oils containing eugenol: A mechanistic viewpoint.. Crit. Rev. Microbiol..

[30] Massoud F., Gauthier S. (2010). Update on the pharmacological treatment of Alzheimer’s disease.. Curr. Neuropharmacol..

[31] Masters C.L., Bateman R., Blennow K., Rowe C.C., Sperling R.A., Cummings J.L. (2015). Alzheimer’s disease.. Nat. Rev. Dis. Primers.

[32] Mikuła-Pietrasik J., Kuczmarska A., Rubiś B., Filas V., Murias M., Zieliński P., Piwocka K., Książek K. (2012). Resveratrol delays replicative senescence of human mesothelial cells *via* mobilization of antioxidative and DNA repair mechanisms.. Free Radic. Biol. Med..

[33] Mollica A., Costante R., Stefanucci A., Pinnen F., Lucente G., Fidanza S., Pieretti S. (2013). Antinociceptive profile of potent opioid peptide AM94, a fluorinated analogue of biphalin with non-hydrazine linker.. J. Pept. Sci..

[34] Mollica A., Pelliccia S., Famiglini V., Stefanucci A., Macedonio G., Chiavaroli A., Orlando G., Brunetti L., Ferrante C., Pieretti S., Novellino E., Benyhe S., Zador F., Erdei A., Szucs E., Samavati R., Dvorácskó S., Tomboly C., Ragno R., Patsilinakos A., Silvestri R. (2017). Exploring the first Rimonabant analog-opioid peptide hybrid compound, as bivalent ligand for CB1 and opioid receptors.. J. Enzyme Inhib. Med. Chem..

[35] Mount C., Downton C. (2006). Alzheimer disease: Progress or profit?. Nat. Med..

[36] Nasar M.Q., Zohra T., Khalil A.T., Ovais M., Ullah I., Ayaz M., Zahoor M., Shinwari Z.K. (2023). Extraction optimization, total phenolic-flavonoids content, HPLC-DAD finger printing, antimicrobial, antioxidant and cytotoxic potentials of Chinese folklore Ephedra intermedia Schrenk & CA Mey.. Bra. J. Pharma. Sci..

[37] Nenadis N., Wang L.F., Tsimidou M., Zhang H.Y. (2004). Estimation of scavenging activity of phenolic compounds using the ABTS(*+) assay.. J. Agric. Food Chem..

[38] Pákáski M., Kálmán J. (2008). Interactions between the amyloid and cholinergic mechanisms in Alzheimer’s disease.. Neurochem. Int..

[39] Prasad S.N. (2013). Muralidhara, Neuroprotective efficacy of eugenol and isoeugenol in acrylamide-induced neuropathy in rats: Behavioral and biochemical evidence.. Neurochem. Res..

[40] Pushpalatha B., Venumadhav N., Swathi M., Raju B. (2013). Neuroprotective effect of resveratrol against scopolamine-induced cognitive impairment and oxidative stress in rats.. Arch. Biol. Sci..

[41] Renner U.D., Oertel R., Kirch W. (2005). Pharmacokinetics and pharmacodynamics in clinical use of scopolamine.. Ther. Drug Monit..

[42] Sarnyai Z., Sibille E.L., Pavlides C., Fenster R.J., McEwen B.S., Tóth M. (2000). Impaired hippocampal-dependent learning and functional abnormalities in the hippocampus in mice lacking serotonin 1A receptors.. Proc. Natl. Acad. Sci..

[43] Sethi S., Joshi A., Arora B., Bhowmik A., Sharma R.R., Kumar P. (2020). Significance of FRAP, DPPH, and CUPRAC assays for antioxidant activity determination in apple fruit extracts.. Eur. Food Res. Technol..

[44] Shoji H., Miyakawa T. (2021). Effects of test experience, closed-arm wall color, and illumination level on behavior and plasma corticosterone response in an elevated plus maze in male C57BL/6J mice: A challenge against conventional interpretation of the test.. Mol. Brain.

[45] Singh R.K. (2020). Recent trends in the management of Alzheimer’s disease: Current therapeutic options and drug repurposing approaches.. Curr. Neuropharmacol..

[46] Stefanucci A., Dimmito M.P., Zengin G., Luisi G., Mirzaie S., Novellino E., Mollica A. (2019). Discovery of novel amide tripeptides as pancreatic lipase inhibitors by virtual screening.. New J. Chem..

[47] Sueishi Y., Ishikawa M., Yoshioka D., Endoh N., Oowada S., Shimmei M., Fujii H., Kotake Y. (2012). Oxygen radical absorbance capacity (ORAC) of cyclodextrin-solubilized flavonoids, resveratrol and astaxanthin as measured with the ORAC-EPR method.. J. Clin. Biochem. Nutr..

[48] Swonger A.K., Rech R.H. (1972). Serotonergic and cholinergic involvement in habituation of activity and spontaneous alternation of rats in a maze.. J. Comp. Physiol. Psychol..

[49] Tong X., Li X., Ayaz M., Ullah F., Sadiq A., Ovais M., Shahid M., Khayrullin M., Hazrat A. (2021). Neuroprotective studies on Polygonum hydropiper L. essential oils using transgenic animal models.. Front. Pharmacol..

